# Golgi structure formation, function, and post-translational modifications in mammalian cells

**DOI:** 10.12688/f1000research.11900.1

**Published:** 2017-11-27

**Authors:** Shijiao Huang, Yanzhuang Wang

**Affiliations:** 1 Department of Molecular, Cellular and Developmental Biology, University of Michigan, Ann Arbor, MI, USA

**Keywords:** Golgi apparatus, structure, formation, cell cycle, Golgi membrane dynamics

## Abstract

The Golgi apparatus is a central membrane organelle for trafficking and post-translational modifications of proteins and lipids in cells. In mammalian cells, it is organized in the form of stacks of tightly aligned flattened cisternae, and dozens of stacks are often linked laterally into a ribbon-like structure located in the perinuclear region of the cell. Proper Golgi functionality requires an intact architecture, yet Golgi structure is dynamically regulated during the cell cycle and under disease conditions. In this review, we summarize our current understanding of the relationship between Golgi structure formation, function, and regulation, with focus on how post-translational modifications including phosphorylation and ubiquitination regulate Golgi structure and on how Golgi unstacking affects its functions, in particular, protein trafficking, glycosylation, and sorting in mammalian cells.

## Overview of Golgi structure and dynamics during the cell cycle

The Golgi apparatus is a central membrane organelle that functions as the post-translational modification factory and trafficking hub for proteins and lipids in the cell. Newly synthesized proteins and membrane lipids enter the endoplasmic reticulum (ER) for proper folding and initial *N*- and *O*-glycosylation and are then transported to the Golgi for trafficking, glycan maturation, and sorting. Each Golgi stack is formed by five to eight tightly aligned flattened cisternae, which can be classified as three separate modules: the *cis*-Golgi network, which is close to the ER and receives the ER output, the stacked *cis*-, *medial*-, and *trans*-Golgi cisternae that contain glycosylation enzymes and process cargo proteins and lipids, and the *trans*-Golgi network (TGN) that is facing the plasma membrane and sorts cargo molecules for delivery to different destinations. In mammalian cells, multiple Golgi stacks are often laterally linked by tubular structures to form a Golgi ribbon.

The Golgi is highly dynamic during the cell cycle, with a unique process of disassembly in early mitosis and reassembly in late mitosis and early interphase ^1^. At the onset of mitosis, the Golgi is sequentially disassembled by ribbon unlinking, cisternae unstacking, and vesiculation, producing vesicles and tubular structures that are dispersed in the cytoplasm ^2^. This is expected to facilitate equal partitioning of the Golgi membranes into the two daughter cells, where the Golgi fragments are reassembled into new cisternae, stacks, and ribbons in late mitosis ^2^. Many proteins, including mitotic kinases and phosphatases, vesicle budding and fusion machineries, Golgi matrix and stacking proteins, soluble *N*-ethylmaleimide-sensitive factor (NSF) activating protein receptors (SNAREs), and membrane fusion proteins, are involved in the Golgi disassembly and reassembly processes during the cell cycle ^3– 11^.

**Figure 1.**
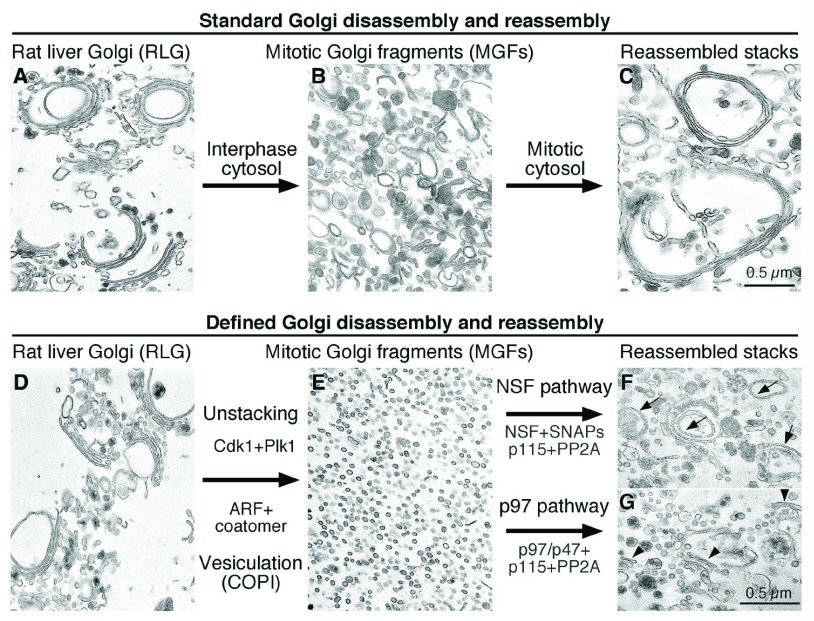
*In vitro* reconstitution of the Golgi disassembly and reassembly processes during the cell cycle. Shown are representative electron micrographs of Golgi membranes during the reactions. **A**– **C**: standard Golgi disassembly and reassembly assay. Purified rat liver Golgi stacks (RLG, **A**) disassembled when treated with mitotic cytosol (MC, **B**) and reassembled when further treated with interphase cytosol (IC, **C**). **D**– **G**: defined Golgi disassembly and reassembly assay. Purified Golgi stacks ( **D**) were fragmented when incubated with cyclin-dependent kinase 1 (Cdk1) and polo-like kinase 1 (Plk1) kinases and ADP ribosylation factor (ARF)/coatomer ( **E**). New Golgi stacks were reassembled from the fragments when further treated with purified proteins in either the *N*-ethylmaleimide-sensitive factor (NSF) ( **F**) or valosin-containing protein (p97) pathway ( **G**). Note that the ubiquitination system was not included in the reaction in ( **G**) and so stacking appeared to be normal but membrane fusion was reduced. Adapted and modified from 6, 14. PP2A, protein phosphatase 2A; SNAP, soluble *N*-ethylmaleimide-sensitive factor attachment protein.

Our current understanding of the regulatory mechanisms of Golgi structure formation during the cell cycle has mostly benefited from an *in vitro* reconstitution assay, which replicates the Golgi disassembly and reassembly processes in the test tube ^12^. By sequential treatment of Golgi membranes purified from rat liver with mitotic cytosol and interphase cytosol prepared from HeLa cells, the Golgi membranes can be disassembled to mitotic Golgi fragments (MGFs) and reassembled into intact Golgi stacks ( Figure 1A–C) ^13, 14^. Subsequent substitution of cytosol with biochemically purified proteins allowed the identification of the minimal machineries and key components that control mitotic Golgi disassembly and post-mitotic Golgi reassembly ^15^. Mitotic disassembly is mediated by cisternal unstacking and vesiculation. Mitotic kinases, including cyclin-dependent kinase 1 (Cdk1), polo-like kinase-1 (Plk1), and perhaps others, phosphorylate the Golgi stacking proteins, membrane tethers, and maybe other unidentified targets, leading to the Golgi reassembly stacking protein of 65 kDa (GRASP65) deoligomerization and Golgi unstacking ^5, 7, 8, 16– 19^. ADP-ribosylation factor 1 (ARF1)-GTP and coatomer vesiculate the cisternae through coat protein I (COPI) vesicle budding ^6, 20^. Treatment of Golgi membranes with a combination of kinases and coat proteins results in complete Golgi fragmentation ( Figure 1D and 1E) ^14, 15^. Post-mitotic Golgi reassembly involves membrane fusion to generate single cisternae, which subsequently form stacks. Fusion is mediated by two ATPases associated with diverse cellular activities (AAAs), NSF and p97 (valosin-containing protein [VCP]; Cdc48 in yeast), each with specific adaptor proteins ( Figure 1F and 1G) ^10, 11, 21– 27^. Restacking occurs through GRASP65 dephosphorylation by the protein phosphatase PP2A ^15^. This *in vitro* reconstitution assay revealed that reversible post-translational modifications, such as phosphorylation and ubiquitination, play essential roles in regulating Golgi membrane dynamics in the mammalian cell cycle ^25, 26, 28– 33^, as discussed below.

## Regulation of Golgi membrane dynamics by phosphorylation during the cell cycle

**Figure 2.**
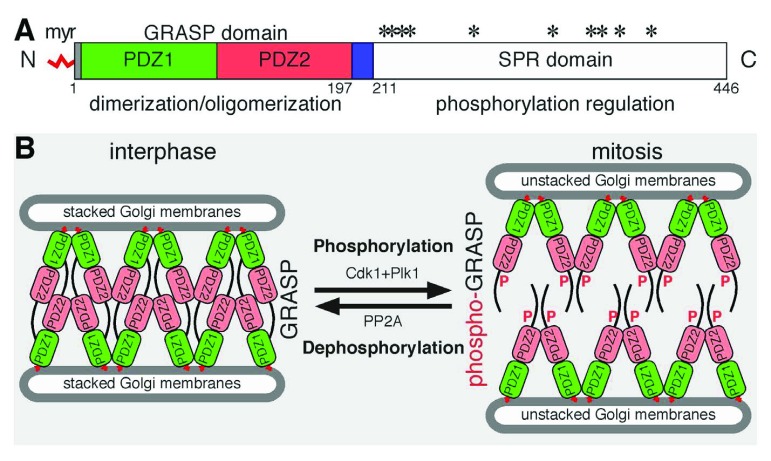
Golgi reassembly stacking protein of 65 kDa (GRASP65) domain structure ( **A**), function, and regulation by phosphorylation ( **B**). **A**: GRASP65 domain structure. Indicated are the myristic acid (myr) for membrane association, the GRASP domain, the two PDZ domains within it for dimerization and oligomerization, and the serine/proline rich (SPR) domain with phosphorylation sites (*). The GM130-binding site is indicated in blue. **B**: GRASP oligomerization and Golgi stack formation. During interphase, GRASP65 homodimers from two neighboring cisternae *trans*-oligomerize through the GRASP domain to “glue” the adjacent cisternae into Golgi stacks. Based on the structure reported by Feng *et al*. ^54^, GRASP *trans*-oligomerization is mediated by PDZ2–PDZ2 interactions, while PDZ1 may interact with the C-terminal unstructured region. In mitosis, GRASP65 is phosphorylated by cyclin-dependent kinase 1 (Cdk1) and polo-like kinase 1 (Plk1) on multiple phosphorylation sites in the SPR domain, which leads to the disassembly of GRASP65 oligomers to homodimers and subsequent Golgi stack disassembly. In late mitosis, GRASP65 is dephosphorylated by protein phosphatase PP2A, reforms oligomers, and restacks Golgi cisternae. Adapted and modified from 33, 46, 51, 54>.

Phosphorylation regulates Golgi stack formation through Golgi stacking proteins. Two peripheral membrane proteins, Golgi reassembly stacking protein of 55 kDa (GRASP55) and GRASP65, share similar domain structures and overlapping functions ^34, 35^. They form mitotically regulated *trans*-oligomers that act as an adhesive “glue” to stick adjacent Golgi cisternae into a stack ( Figure 2) ^7, 16^. GRASP65 is predominantly concentrated in the *cis*-Golgi, while GRASP55 is localized in the *medial-trans*-cisternae ^35^. Inhibition of GRASP65 or GRASP55 on MGFs by antibodies reduces post-mitotic stacking of newly formed cisternae in the *in vitro* Golgi reassembly assay ^34, 35^. Microinjection of antibodies against GRASP65 or GRASP55 into mitotic cells inhibits Golgi reassembly in newly divided daughter cells ^7, 36^. Depletion of either GRASP65 or GRASP55 by RNA interference (RNAi) reduces the number of cisternae per stack, while simultaneous depletion of both GRASPs results in complete disassembly of the Golgi ^8, 37^. The role of GRASP55/GRASP65 in Golgi stacking has been confirmed in cells in which GRASP55 and/or GRASP65 are knocked out by the clustered regularly interspaced short palindromic repeats (CRISPR)/cas9 genome editing technique ^38^. Double knockout of GRASP proteins disperses the Golgi stack into single cisternae and tubulovesicular structures ^38^. In another report ^39^, simultaneous depletion of both GRASPs and their interacting golgins, golgin-45 and GM130, is required for complete destruction of the Golgi stack, suggesting that other proteins, such as golgins, may also contribute to Golgi stack formation.

In addition to stacking, GRASP55 and GRASP65 have also been shown to link Golgi stacks into a ribbon ^38, 40– 42^. RNAi-mediated depletion of GRASP65 or GRASP55 also resulted in Golgi ribbon unlinking ^40, 41, 43^, suggesting a possibility that GRASPs may function in both Golgi stacking and ribbon linking by forming *trans*-oligomers. Given that the gaps between Golgi stacks are much larger and more heterogeneous (tens to hundreds of nanometers) than the distance between cisternae within each stack ^44^, it is possible that other bridging proteins may help GRASPs in ribbon linking. Indeed, a number of GRASP-interacting proteins have been recently identified and characterized; many of them are involved in Golgi structure formation. One example is the actin elongation factor Mena ^45^. Mena interacts with GRASP65 and thus is recruited onto the Golgi membranes. Knockdown of Mena or disruption of actin polymerization leads to Golgi fragmentation. In cells, Mena and actin are required for Golgi ribbon formation after nocodazole washout; *in vitro,* Mena and microfilaments enhance GRASP65 oligomerization and Golgi membrane fusion. Therefore, Mena’s interaction with GRASP65 promotes local actin polymerization, which in turn enables Golgi ribbon linking ^45^. Taken together, these results demonstrate that GRASP proteins play important roles in Golgi structure formation.

Each GRASP protein comprises an N-terminal GRASP domain that forms dimers and oligomers and a C-terminal serine/proline-rich (SPR) domain that contains multiple phosphorylation sites ( Figure 2A) ^46^. GRASP proteins form *trans*-oligomers through the GRASP domain, which is regulated by phosphorylation in the C-terminal SPR domain ( Figure 2B) ^16^. In mitosis, GRASP65 is phosphorylated by mitotic kinases Cdk1 and Plk1 on multiple phosphorylation sites in the SPR domain, which leads to the disassembly of GRASP65 oligomers and subsequent disassembly of the Golgi stack ^29, 47^. In late mitosis, GRASP65 is dephosphorylated by PP2A, reforms oligomers, and restacks Golgi cisternae ( Figure 2) ^15^. Similarly, GRASP55 oligomerization and function are also regulated by phosphorylation, as GRASP55 is phosphorylated by MEK1/ERK kinases during mitosis; however, the phosphatase that dephosphorylates GRASP55 in later mitosis is unknown ^8, 48^. Expression of non-phosphorylatable mutants of GRASP55/GRASP65 increases the number of cisternae per stack in interphase and inhibits Golgi disassembly in mitosis ^8, 16, 37^. Golgi ribbon linking by GRASPs is also regulated by mitotic phosphorylation. It has been shown that phosphorylation of GRASP55 by ERK blocks Golgi ribbon linking ^41^, while in another report GRASP65 phosphorylation by Plk1 on Ser189 is required for Golgi ribbon unlinking in mitosis ^49^. In summary, the reversible phosphorylation of Golgi stacking proteins is a key regulatory mechanism of mitotic Golgi disassembly and post-mitotic Golgi reassembly.

The GRASP domain of GRASP55 and GRASP65 contains two PDZ domains, whose interactions enable the homo-dimerization and oligomerization of the GRASP proteins ( Figure 2) ^37, 50, 51^. Details on the PDZ interactions as well as GRASP dimerization and oligomerization have been revealed by the crystal structures of GRASP55 and GRASP65; however, there are some discrepancies in the GRASP structures reported by three different research groups ^51^. In the structure reported by Truschel *et al*. ^52, 53^, GRASPs form antiparallel *trans*-oligomers through PDZ1–PDZ2 interactions. In the structure reported by Feng *et al*. ^54^, GRASP *trans*-oligomerization is mediated by PDZ2–PDZ2 interactions, while PDZ1 may interact with the C-terminal unstructured region of the molecule ( Figure 2). A third structure by Shi and coworkers revealed the interaction between GRASPs and their interacting golgins, e.g. GRASP55 with a Golgin-45 peptide ^55^ and GRASP65 with a GM130 peptide ^56^, and the structure is largely consistent with the structure reported by Feng *et al*. ^54^. The discrepancies in the reported structures of the GRASP proteins can be possibly explained by the fact that GRASPs are intrinsically disordered proteins, as revealed by bioinformatic and biophysical analysis of the GRASP55/GRASP65 homologue in *Cryptococcus neoformans* (CnGRASP) ^57^. Perhaps such a property with variable conformations may allow GRASPs to perform a large array of functions by forming homodimers and oligomers and by interacting with multiple interacting partners. It is worth mentioning that oligomerization of GRASP65 is regulated by mitotic phosphorylation of the C-terminal SPR domain, while dimerization is not ^7, 16^; however, none of the reported crystal structures contain the unstructured C-terminal half of the GRASP molecules, and thus the structural basis for phosphorylation-mediated regulation of GRASP oligomerization is currently unavailable.

Phosphorylation also regulates Golgi disassembly and reassembly through modulating membrane tethering and fusion. The Golgi is the trafficking center in the vesicular trafficking pathway. Vesicular transport includes three steps: vesicle budding from a donor membrane, vesicle transport and tethering, and vesicle fusion with the target membrane. In interphase, COPI vesicles bud from the Golgi membranes and fuse with the acceptor membranes for retrograde intra-Golgi and Golgi-to-ER trafficking ^58^. COPI vesicle tethering on the targeting membranes is facilitated by the interaction between *cis*-Golgi matrix protein GM130 and the tethering factor p115, which facilitates vesicle fusion with the targeting membranes mediated by SNARE proteins ^4^. During mitosis, GM130 is phosphorylated by Cdk1, which prevents GM130–p115 interaction and thus inhibits vesicle tethering and subsequent membrane fusion ^4, 9, 28^. Consequently, the Golgi is fragmented in mitosis because of continuous budding of COPI vesicles while membrane fusion is inhibited. At the end of mitosis, GM130 is dephosphorylated by PP2A, which restores the GM130–p115 interaction and membrane fusion ^9^. Several other golgins, including golgin-67 ^59^, golgin-84 ^60^, golgin-160 ^61^, and p115 ^62^, may also be regulated by phosphorylation in a similar manner as GM130 ^63, 64^.

The exact role of mitotic Golgi disassembly is so far not fully understood. In addition to facilitating equal partitioning of Golgi membranes into the two daughter cells ^65^, it may also function as a cell cycle checkpoint to allow mitotic progression ^36, 66– 74^. Mechanistically, Golgi disassembly is required for proper assembly of mitotic spindle ^73^. Spindle assembly is regulated by the spindle assembly factor TPX2, which is normally sequestered by the interaction with importin α. At the onset of mitosis, GM130 binds and sequesters importin α to the Golgi membranes, releasing the activity of TPX2 for spindle assembly in the vicinity of Golgi membranes. GM130 then captures the nascent microtubules and thus couples the Golgi membranes to the forming spindle ^73^. Emerging evidence shows that GM130 is also involved in autophagy. Under growth conditions, GM130 tethers GABARAP to the Golgi and inhibits autophagy. Upon amino acid starvation, another GM130-interacting protein, WAC, interacts with GM130 and suppresses the binding of GABARAP to GM130. This allows the relocalization of GABARAP to centrosomes, which facilitates autophagosome formation ^75^. So far, it is not known whether phosphorylation regulates GM130 in mitotic spindle assembly and autophagy. In summary, phosphorylation regulates Golgi membrane dynamics during cell division: mitotic phosphorylation of GRASP proteins impairs GRASP oligomerization and causes Golgi ribbon unlinking and cisternal unstacking, while phosphorylation of membrane tethers results in vesiculation of Golgi membranes. Further studies will determine whether phosphorylation also regulates GRASP-interacting proteins.

## Regulation of Golgi membrane dynamics by monoubiquitination during the cell cycle

In addition to phosphorylation, monoubiquitination also regulates Golgi membrane dynamics through modulating membrane fusion. Membrane fusion is a key factor that determines Golgi disassembly and reassembly during the cell cycle. All membrane fusion events in the intracellular trafficking pathways and biogenesis of membranous organelles in the endomembrane system are mediated by SNARE proteins ^76– 78^. SNARE proteins are characterized by an evolutionarily conserved coiled-coil SNARE domain of about 60 to 70 amino acids. Most SNARE proteins are anchored on membranes by a C-terminal transmembrane domain ^76^. To mediate membrane fusion, one v-SNARE from the vesicle membrane and three t-SNAREs from the target membrane bundle through the α helices of the SNARE domain and zipper up to form a *trans*-SNARE complex that pulls the opposing membranes to close proximity for fusion ^79^. After membrane fusion, the *trans*-SNARE complex transits to *cis*-SNARE complex, which is subsequently disassembled by the AAA ATPase, NSF, powered by ATP hydrolysis. NSF and its cofactors, α/γ-SNAPs (soluble NSF attachment proteins), are required for membrane fusion by mediating SNARE complex disassembly and SNARE protein dissociation to recycle SNARE proteins for the next round of membrane fusion ^80^. Post-mitotic Golgi membrane fusion requires two AAA ATPases, NSF and p97, as described above. NSF-mediated post-mitotic Golgi membrane fusion requires the Golgi t-SNARE syntaxin 5 and its cognate v-SNARE GS28 ^11^. The other AAA ATPase, p97, and its adaptor protein, p47, promote post-mitotic Golgi membrane fusion independently and non-additively to NSF-mediated post-mitotic membrane fusion. The p97/p47 pathway does not require the tethering factor p115 or the v-SNARE GS28 but shares a common Golgi t-SNARE, syntaxin 5, with the NSF/SNAP pathway ^11^.

A major difference between p97/p47- and NSF/SNAP-mediated post-mitotic membrane fusion is the involvement of ubiquitin in the p97/p47 pathway ^31^. Initially, the lack of membrane fusion activity in the *in vitro* Golgi reassembly reaction with purified p97/p47 ( Figure 1G) prompted us to look into the involved proteins more carefully. The first hint that ubiquitin is involved in p97/p47-mediated fusion is that p47 contains a ubiquitin-associated (UBA) domain, which preferably binds to monoubiquitin over polyubiquitin ^31^. Interruption of the interaction between p47 and ubiquitin inhibits p97/p47-mediated post-mitotic Golgi membrane fusion. Monoubiquitin binds the UBA domain of p47 and recruits the p97/p47 membrane fusion machinery to the MGFs ^31^. Proteasome activity is not involved in either Golgi disassembly or reassembly ^26^. These results indicate that monoubiquitin is involved in p97/p47-mediated membrane fusion. The E3 ligase and the deubiquitinase involved in Golgi disassembly and reassembly were then identified. Ubiquitination is mediated by a Golgi-localized E3 ligase HACE1 (the HECT domain and ankyrin repeat containing E3 ubiquitin protein ligase 1), which occurs during the Golgi disassembly process in mitosis but is required for the subsequent Golgi reassembly ^25^. The p97/p47-binding protein VCIP135 (VCP [p97]/p47 complex-interacting protein, p135) functions as a deubiquitinating enzyme whose activity is required for post-mitotic Golgi reassembly ^26^. The deubiquitinase activity of VCIP135 is inactivated in metaphase by Cdk1-mediated phosphorylation on the S130 residue and reactivated in telophase upon dephosphorylation ^30, 81, 82^.

**Figure 3.**
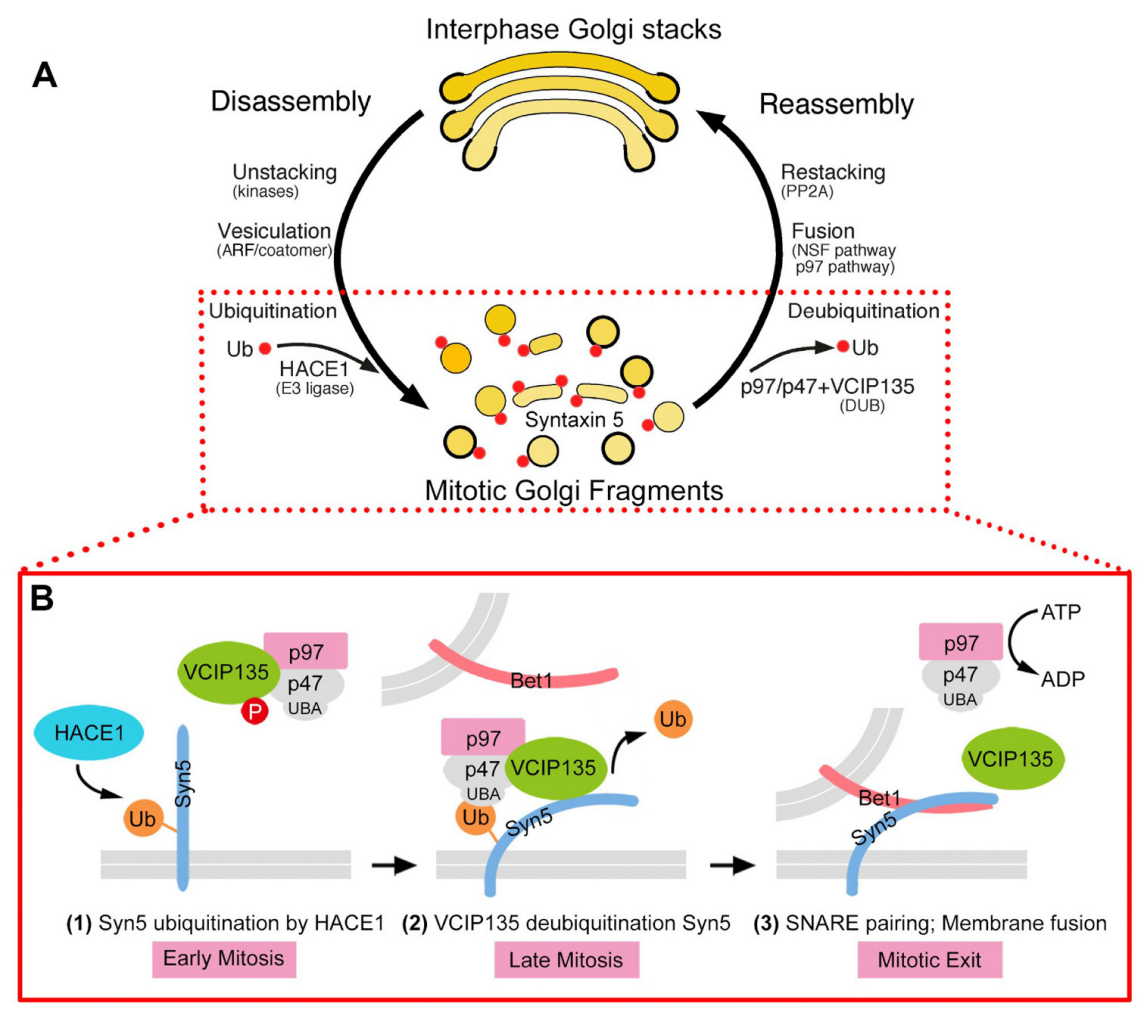
Monoubiquitination of syntaxin 5 regulates p97/p47-mediated post-mitotic Golgi membrane fusion during the cell cycle. **A**: Interphase Golgi stacks are disassembled by cisternal unstacking and vesiculation. Mitotic kinases phosphorylate the stacking proteins, leading to Golgi reassembly stacking protein (GRASP) deoligomerization and Golgi unstacking. ADP-ribosylation factor 1 (ARF1)-GTP and coatomer vesiculate the cisternae through COPI vesicle budding. Post-mitotic Golgi reassembly involves membrane fusion to generate single cisternae that subsequently form stacks. Membrane fusion is mediated by two AAA ATPases, *N*-ethylmaleimide-sensitive factor (NSF) and valosin-containing protein (p97), with their adaptor proteins. **B**. Monoubiquitination of syntaxin 5 (Syn5) regulates p97-mediated post-mitotic Golgi membrane fusion. In early mitosis, Syn5 is monoubiquitinated by an E3 ligase, the HECT domain and ankyrin repeat containing E3 ubiquitin protein ligase 1 (HACE1), on the Golgi membranes, while the deubiquitinase p97/p47 complex-interacting protein, p135 (VCIP135) is inactivated by mitotic phosphorylation ( **1**). Ubiquitinated Syn5 recruits the p97/p47 complex through the ubiquitin-associated (UBA) domain of p47 to the Golgi membranes ( **2**). In late mitosis, Syn5 is deubiquitinated by VCIP135 that is reactivated by dephosphorylation, which enables Syn5–Bet1 soluble NSF activating protein receptor (SNARE) complex formation and thus membrane fusion by p97 at mitotic exit ( **3**). Adapted and modified from 32. PP2A, protein phosphatase 2A, Ub, ubiquitin.

Recently, the ubiquitination substrate on the Golgi membranes for HACE1 and VCIP135 has been identified as the Golgi t-SNARE syntaxin 5. Syntaxin 5 is monoubiquitinated in early mitosis by HACE1 on lysine 270 within the SNARE domain, which prevents the interaction between syntaxin 5 and Bet1 and blocks SNARE complex formation and membrane fusion, resulting in Golgi fragmentation in mitosis. On the other hand, ubiquitin on syntaxin 5 recruits the p97/p47 membrane fusion machinery to the mitotic Golgi membranes and facilitates post-mitotic Golgi membrane fusion ^32^. In late mitosis, VCIP135 deubiquitinase activity is reactivated, which removes the monoubiquitin on syntaxin 5 and promotes SNARE complex formation and post-mitotic Golgi membrane fusion ^32^. These results reveal that cycles of addition and removal of ubiquitin to and from substrates regulate Golgi membrane dynamics during cell division ( Figure 3). Together with reversible phosphorylation in mitosis, reversible monoubiquitination regulates Golgi disassembly and reassembly during the cell cycle by blocking membrane fusion in mitosis and facilitating membrane fusion at mitotic exit.

## Golgi defects in diseases

Golgi structural defects have been observed in many diseases such as Smith-McCort dysplasia ^83^, MACS (macrocephaly, alopecia, cutis laxa, and scoliosis) syndrome ^84, 85^, Alzheimer’s disease (AD) ^86– 88^, Parkinson’s disease (PD) ^89^, Huntington’s disease (HD) ^90^, and amyotrophic lateral sclerosis (ALS) ^91– 93^. Golgi dysfunction and protein trafficking defects have been reported in Pelizaeus-Merzbacher disease ^94^, proximal spinal muscular atrophy ^95^, and dyschromatosis universalis hereditaria ^96^. In addition, Golgi glycosylation defects have been linked to Angelman syndrome ^97^ and cutis laxa type II and wrinkly skin syndrome ^98, 99^. Defects in the conserved oligomeric Golgi (COG) complex, a membrane tether involved in retrograde trafficking of Golgi glycosylation enzymes, have been reported to cause human Congenital Disorders of Glycosylation (CDG), which are rare genetic diseases with defective *N*-glycan and *O*-glycan biosynthesis ^100^. Glycosylation defects have been linked to the pathogenesis of diabetes ^101^, cancer ^102^, and cystic fibrosis ^103, 104^.

Because of the broad functions that glycans and glycoproteins play, it is important that glycosylation is reliable and accurate. However, compared to that of protein and DNA, the synthesis of glycan polymers follows no template, and an estimated 700 proteins are required for producing the varied glycan structures with high fidelity, including glycosyltransferases (addition of sugars), glycosidases (removal of sugars), and nucleotide sugar transporters (supply of sugar substrates) ^105^. Therefore, the process of protein glycosylation needs to be highly organized and sequential. As the main station of sugar chain maturation, the Golgi must use every element to safeguard this highly efficient enzymatic event, and, undoubtedly, Golgi defects may lead to diseases by affecting the accuracy of glycosylation. The exact mechanisms that cause Golgi defects and the contribution of Golgi defects to disease development are largely unexplored and may vary between diseases but have started to draw the attention of Golgi cell biologists.

One example of such a study is on Golgi defects in AD. Using mouse and cell models that express the human Swedish mutant of the amyloid precursor protein (APPswe, KM 593/594 NL) and the exon 9 deletion mutant of human presenilin-1 (PS1ΔE9), Joshi *et al*. ^88^ revealed that the accumulation of amyloid beta (Aβ) peptides leads to Golgi fragmentation. Mechanistically, Aβ accumulation triggers Cdk5 activation, which phosphorylates GRASP65 and causes Golgi fragmentation. Subsequently, Golgi fragmentation accelerates APP trafficking and increases Aβ production ^88^. Significantly, rescuing Golgi structure by suppressing Cdk5 activation and thus inhibiting GRASP65 phosphorylation, or by expressing nonphosphorylatable GRASP65 mutants, both reduce Aβ secretion by elevating α-cleavage of APP ^88^. Given the important roles of the Golgi in protein trafficking and processing, Golgi defects may perturb the proper trafficking and processing of many essential neuronal proteins, resulting in compromised neuronal function and neuronal death. Alternatively, GRASP65 may directly regulate APP trafficking and processing, as GRASP65 has been reported to directly control the trafficking of several transmembrane proteins including TGF-α, CD8-α, and p24 cargo receptor family proteins ^106– 108^. A common feature of these proteins, as discussed below, is that they all contain a valine residue at the C-terminal cytoplasmic tail, which is critical for their interaction with GRASP proteins. However, APP does not contain a C-terminal valine, and so far no evidence has been provided to support a direct role for GRASP55/GRASP65 in APP trafficking. Overall, this study provides a molecular mechanism for Golgi fragmentation and its effects on APP trafficking and processing in AD, suggesting the Golgi as a potential drug target for AD treatment ^88, 109, 110^. Similar mechanisms may apply to some other neurodegenerative diseases with Golgi defects.

## Consequence of Golgi defects on trafficking, sorting, and glycosylation

Golgi cisternae do not usually form stacks in the budding yeast *Saccharomyces cerevisiae*, implying that stacking is not necessary for cell survival. However, Golgi stacking is a prominent feature in all metazoans and many unicellular eukaryotes, suggesting that it has important effects on Golgi function. It has been known for decades that the Golgi membranes form a stacked structure; however, it has been a mystery why Golgi structure formation is essential for its function in protein trafficking and processing. This was largely owing to the lack of molecular tools that can be used to modulate Golgi structure formation. The identification of the Golgi stacking proteins GRASP55 and GRASP65 allowed the manipulation of Golgi stack formation and thus the determination of the biological significance of Golgi structure formation. As expected, double knockdown or knockout of GRASP55 and GRASP65 leads to the disassembly of the Golgi stack but does not cause cell death; rather, it impairs Golgi functions in protein trafficking, sorting, and glycosylation ^38, 111^, as discussed below.

Destruction of the Golgi stacks by GRASP inhibition accelerates protein trafficking but causes protein missorting. The Golgi is the transit center for protein trafficking and sorting for delivery to different destinations, including incorporation into membrane organelles, secretion to extracellular space, or degradation in lysosomes. Hypothetically, the close spatial arrangement of cisternae in stacks minimizes the distance that molecules must travel. Local tethering proteins facilitate vesicle fusion with Golgi membranes ^112^; therefore, stacking should enhance protein trafficking. However, experimentally, disruption of Golgi stack formation by inhibiting GRASP functions resulted in the opposite effects. Inhibition of Golgi stack formation by microinjection of GRASP65 antibodies accelerates CD8 trafficking ^5, 39^. Depletion of both GRASPs leads to the destruction of the entire Golgi architecture and enhances trafficking of the cell adhesion protein integrin, the vesicular stomatitis virus G glycoprotein (VSVG), and the lysosomal enzyme cathepsin D ^38, 111^. Golgi destruction increases the rate and efficiency of COPI vesicle formation *in vitro* ^5^ and membrane association of coat proteins in cells ^111^. Golgi destruction also causes missorting of the cathepsin D precursor to the extracellular space ^111^, suggesting that stacking may ensure that sorting occurs only when cargo molecules reach the TGN but not in earlier subcompartments of the Golgi.

It is worth mentioning that GRASPs have been implicated in the transport of specific cargo proteins, such as TGFα ^106^, p24 ^108^, CD83 ^113^, CD8α, and Frizzled4 ^107^. These proteins contain a C-terminal hydrophobic cytoplasmic tail in which a critical valine residue interacts with the PDZ domain of the GRASP proteins. Here, GRASPs function as cargo receptors or chaperones for these transmembrane proteins. Therefore, depending on the type of cargo, GRASP depletion may have different effects. For the transmembrane proteins with a C-terminal valine, knockdown of GRASP proteins may slow down trafficking, whereas for other cargo proteins without such a signature, such as VSV-G, integrin, and cathepsin D, an opposite effect is expected ^5, 39, 111^. GRASPs are also involved in unconventional secretion, a pathway that bypasses the Golgi, as extensively discussed elsewhere ^114– 116^.

Golgi destruction also impairs accurate protein glycosylation. Glycosylation in the Golgi lumen can be divided into two types: *N*-linked glycosylation, by which the oligosaccharides are linked to the amide nitrogen of asparagine, and *O*-linked glycosylation, by which the oligosaccharides are linked to the hydroxyl group of serine, threonine, hydroxylysine, or tyrosine. The Golgi is the main station for protein oligosaccharide processing and maturation, and it harbors various glycosyltransferases and glycosidases in different subcompartments; an ordered structure is likely required to carry out precise, sequential modifications as cargo proteins pass between cisternae ^117– 119^. In budding yeast, *N*-glycosylation in the Golgi mainly involves the addition of mannoses ^120^. In multicellular organisms, *N*-glycosylation of membrane and secretory proteins is more complex and critical. Accurate glycosylation is essential for their cellular functions, including cell adhesion and migration, cell–cell communication, and immunity ^121^. In polarized cells, for example neurons and epithelial cells, *N*- and *O*-linked glycans function as apical sorting signals ^122^, which could be the reason why stacking is not necessary in yeast but is crucial for life in higher-order organisms.

In mammalian cells, Golgi destruction by GRASP depletion decreases glycoprotein glycosylation and glycan complexity without impacting the expression level and localization of Golgi enzymes. A reasonable explanation for the decrease in glycoprotein glycan complexity is that as cargo moves more rapidly through unstacked Golgi compartments induced by GRASP depletion, resident enzymes have less time to recognize and modify their substrates ^111, 123^. As a result, glycoproteins contain more trimmed high-mannose glycans and fewer complex glycan structures in the GRASP knockdown cells. This effect was not due to Golgi ribbon unlinking, as suggested by some other studies ^40^, and was also proven by the fact that the induction of Golgi ribbon unlinking by the depletion of golgin-84 ^60^ and by nocodazole treatment ^124, 125^ did not result in the same effect as GRASP depletion ^111^. Double knockdown or knockout of GRASPs decreases *N*-linked oligosaccharides on the cell surface, with a reduction in both high-mannose and complex-type glycans ^38, 111^. These results demonstrate an intimate link between the structure and function of the Golgi ^46, 123^. Interestingly, GRASP depletion decreased not only *N*-linked glycan complexity but also global *N*-linked glycoprotein glycosylation ^111^. It is not immediately obvious why the total *N*-linked glycan amount should be reduced in GRASP knockdown cells. Since *N*-linked glycoprotein glycosylation is initiated in the ER, we speculate a feedback pathway exists to monitor Golgi processing and adjust the flux of protein trafficking and processing through early secretory compartments such as the ER. In summary, the Golgi stack is an indispensable structure to ensure a proper flux for protein trafficking and accurate glycosylation.

## Concluding remarks

Golgi is the central organelle for the glycosylation, trafficking, and sorting of secretory and membrane proteins and lipids. To perform these complex functions, the Golgi membranes need to form a unique stacked structure and, subsequently, a linked ribbon. This structure undergoes morphological changes under physiological conditions such as during cell cycle progression and under stress conditions such as in diseases. In the last decade or so, much progress has been made in understanding the mechanism of Golgi structure and function. First, the identification of GRASP *trans*-oligomers provides a plausible explanation of how Golgi membranes form stacks and ribbons. GRASPs are the major targets for phosphorylation regulation during the cell cycle and in AD. To better understand GRASP functions and regulation, it is necessary to identify and characterize GRASP-interacting proteins. Second, it is now better known that the Golgi disassembly and reassembly processes during cell division are regulated by reversible protein post-translational modifications, in particular, protein phosphorylation that regulates cisternal stacking through GRASPs and membrane tethering through golgins, and monoubiquitination of the Golgi t-SNARE syntaxin 5 that controls p97/p47-mediated membrane fusion. So far, the cognate SNAREs of syntaxin 5 in post-mitotic Golgi membrane fusion still need to be identified. More work is also needed to figure out whether p97/p47 mediates homotypic or heterotypic membrane fusion of MGFs. Third, we now have a better idea of why Golgi structure formation is required for its function. Golgi structure serves as a quality control mechanism to ensure accurate protein glycosylation and sorting by slowing down protein trafficking. It is generally believed that Golgi structure formation and its morphological changes are adjusted to best perform its functions. More work needs to be done to investigate how Golgi adapts its structure to best fulfill its function under different conditions and what roles GRASPs play in these responses. This is interesting, as it may help understand how Golgi defects contribute to the pathogenesis of diseases.

## Abbreviations

AAA, ATPases associated with diverse cellular activities; Aβ, amyloid beta; AD, Alzheimer’s disease; APP, amyloid precursor protein; Cdk1, cyclin-dependent kinase 1; ER, endoplasmic reticulum; GRASP55, Golgi reassembly stacking protein of 55 kDa; GRASP65, Golgi reassembly stacking protein of 65 kDa; HACE1, the HECT domain and ankyrin repeat containing E3 ubiquitin protein ligase 1; MGF, mitotic Golgi fragment; NSF, *N*-ethylmaleimide-sensitive factor; p97/VCP, valosin-containing protein; Plk1, polo-like kinase 1; RNAi, RNA interference; SNAP, soluble *N*-ethylmaleimide-sensitive factor attachment protein; SNARE, soluble *N*-ethylmaleimide-sensitive factor activating protein receptor; SPR, serine/proline-rich; TGN, *trans*-Golgi network; UBA, ubiquitin-associated; VCIP135, valosin-containing protein (p97)/p47 complex-interacting protein, p135.
